# Analysis of Breakthrough Reactions in 1,143 Desensitization Procedures in a Single Tertiary Hospital Using a One-Bag Desensitization Protocol

**DOI:** 10.3389/falgy.2022.786822

**Published:** 2022-02-11

**Authors:** Hyun Hwa Kim, Jeongmin Seo, Yoon Hae Ahn, Hyunjee Kim, Jeong-Eun Yoon, Jang Ho Suh, Dong Yoon Kang, Suh Young Lee, Hye-Ryun Kang

**Affiliations:** ^1^Drug Safety Center, Seoul National University Hospital, Seoul, South Korea; ^2^Department of Internal Medicine, Seoul National University College of Medicine, Seoul, South Korea; ^3^Institute of Allergy and Clinical Immunology, Seoul National University Medical Research Center, Seoul, South Korea

**Keywords:** drug hypersensitivity, antineoplastic agents, desensitization, breakthrough reaction, premedication

## Abstract

**Background:**

Drug desensitization is helpful for patients who have experienced significant hypersensitivity reactions (HSRs) to antineoplastic agents. One-bag desensitization protocols, attracting attention in recent years, need to be validated on their safety and efficacy in a large number.

**Methods:**

One-bag desensitization procedures conducted from 2018 to 2020 were analyzed; their outcomes and the risk factors for breakthrough reactions (BTRs) were assessed in desensitization procedures to major drug types (platins, taxanes, and monoclonal antibodies).

**Results:**

A total of 1,143 procedures of one-bag desensitization were performed in 228 patients with 99% completion rate. BTRs occurred in 26% of the total desensitization procedures—34% in platins, 12% in taxanes, and 18% in mAbs. BTR occurrence rate decreased along the desensitization process with 80% of BTRs occurring within the 6th desensitization attempts. Severe BTR occurred more frequently with severe initial HSRs (1% in mild to moderate initial HSRs vs. 16% in severe). Severe initial HSR was also a significant risk factor for moderate to severe BTR in platins (odds ratio 1.56, 95% confidence interval [CI] 1.06–2.29, *p* = 0.025). The use of steroid was also associated with lower occurrence of moderate to severe BTR (odds ratio 0.50, 95% CI 0.35–0.72, *p* < 0.001).

**Conclusion:**

Most patients with HSRs to antineoplastic agents can safely receive chemotherapy through a one-bag desensitization protocol. Further studies on each drug with larger sample size can help verify the risk factors of BTRs and evaluate the efficacy of steroid premedication in improving the safety of desensitization in high-risk patients.

## Introduction

All drugs have a potential for causing adverse reactions, and severe drug allergy may inhibit patients from receiving the treatment they need. When serious hypersensitivity reactions (HSRs) appear during the first-line treatment, the use of alternative drugs may be inevitable, which in turn can lead to increased costs and treatment failure.

Desensitization may provide an alternative to switching to second-line treatment as it allows patients with a history of HSRs to receive the culprit drug without triggering an allergic response (1–3). During desensitization, patients receive the medication in incremental doses starting from a small amount and ultimately reaching the target dose. Rapid drug desensitization is indicated in type I HSRs (which are usually immediate, and might show an elevation of serum tryptase regardless whether they are IgE-dependent or non-IgE-dependent), cytokine release reactions (which are usually immediate and show an elevation of interleukin (IL)-6), mixed reactions (featuring characteristics of both type I and cytokine release reactions), and benign mild type IV HSRs (which are usually non-immediate) (4, 5).

In the past, desensitization was mostly done with protocols using stepwise-diluted solutions such as a 3-bag 12-step or a 4-bag 16-step protocol (3, 6, 7). High-precision pumps now allow for desensitization without serial dilution (8). One-bag protocols without dilution have been recently reported with advantages of reduction in time and the risk of potential errors without compromising the safety, efficacy, and tolerability of the process (9–13). We have also designed one-bag desensitization protocols for antineoplastic agents that are non-inferior compared to multi-bag protocols (14–16) and have been applying the standardized one-bag protocol since 2018.

It is important to distinguish patients with higher risks of developing HSRs during chemotherapy or experiencing breakthrough reactions (BTRs) during desensitization. Several studies have reported the risk factors of HSRs which include sex, blood eosinophil count, and a history of drug allergy (1, 5, 17–21). Others have shown that atopy, positive skin prick test, high total IgE titer, and previous exposure to high doses of culprit drugs are associated with higher incidence of BTRs during desensitization (1, 22, 23).

Various agents are used as premedication during desensitization to prevent or alleviate BTRs. H1 receptor antagonists (e.g., diphenhydramine) and H2 receptor antagonists (e.g., famotidine) are the most commonly given forms of premedication (24). Aspirin and montelukast are often added to alleviate symptoms caused by mast cell mediator release (25). For the desensitization of monoclonal antibodies, premedication with acetaminophen can prevent or attenuate cytokine-release related symptoms (24). Steroids exert anti-inflammatory effects through gene expression modulation—a relatively slow process that results in a delayed onset of action (26). As such, whether steroids can prevent immediate reactions caused by mast cell activation remains controversial. Regarding desensitization of antineoplastic agents, the frequent usage of steroids as antiemetics further complicates the evaluation of their efficacy (27). Steroids are included in some published desensitization protocols (28–30), but they are not routinely recommended as premedication.

In this study, we share our experiences of utilizing a one-bag desensitization protocol for various antineoplastic agents and identify the risk factors for BTR occurrence.

## Materials and Methods

### Study Design

This study is a retrospective observational study that included patients who underwent one-bag desensitization protocols for antineoplastic agents in Seoul National University Hospital from January 2018 to December 2020. When HSRs occurred during chemotherapy, the patients were referred to the allergy department, where allergists assessed the patients, performed diagnostic tests such as skin tests if needed, and applied desensitization if eligible. At each admission for chemotherapy, their previous desensitization was evaluated by allergists to tailor their desensitization protocol—modulating the interval and number of steps according to pre-determined algorithms. Desensitization was performed by trained oncology nurses under the supervision of allergists. The drugs included in this study were limited to platins (carboplatin, cisplatin, oxaliplatin), taxanes (docetaxel, paclitaxel), and monoclonal antibodies (mAbs) (brentuximab, cetuximab, daratumumab, infliximab, obinutuzumab, pembrolizumab, pertuzumab, rituximab, trastuzumab). An example of the protocol is shown in Supplementary Table 1.

Prior to desensitization, skin testing was performed to oxaliplatin, carboplatin, cisplatin, paclitaxel, docetaxel, cetuximab, rituximab, and obinutuzumab in some patients. Skin test started with a skin prick test with the undiluted drug shown in Supplementary Table 2. Positive (histamine 10 mg/mL) and negative (saline) controls were used. If skin prick test showed negativity, intradermal testing was done with a 10-fold diluted solution except for paclitaxel in which 100-fold dilution was used. Each test was read in 15 min. The prick test result was considered positive when a wheal of at least 3 mm developed with surrounding flare, whereas the intradermal test result was considered positive if a mean wheal diameter of minimum 3 mm greater than the negative control at the presence of surrounding flare.

### Methods

Electronic medical records of the study subjects were investigated. Baseline data including patients' sex, age, diagnosis, number of chemotherapy cycles, the symptoms and severity of the initial HSR, the result of skin test if performed, number of desensitization cycles, the use of steroid premedication, occurrence of BTRs, and the symptoms and severities of BTRs were gathered. The severity of initial HSRs and BTRs was evaluated as follows: symptoms involving only skin and subcutaneous tissue were classified as mild or grade 1; features suggesting respiratory, cardiovascular, or gastrointestinal involvement were classified as moderate or grade 2; hypoxia, hypotension, or neurologic compromise were considered as severe or grade 3 reactions (31).

The overall BTR occurrence rate (the number of procedures with BTR/the number of all procedures) and desensitization completion rate (the number of procedures which completed administration/the number of all procedures) were calculated across major drug types (platins, taxanes, and mAbs). Desensitization procedures were classified into procedures with no or mild BTRs and those with moderate to severe BTRs, and the severity of initial HSR, number of desensitization cycles, and the use of steroid premedication were compared between the two groups.

### Statistics and Ethics

Categorical variables were presented as frequencies and percentages. The number of desensitization cycles was presented as means and standard deviations. The number of chemotherapy cycles for initial HSR was presented as medians and interquartile ranges. The difference in BTR occurrence rate and completion rate among drug types was analyzed using the ANOVA method. The difference between categorical variables was tested by the chi-square method or Fisher's exact test. Variables were analyzed using a multivariate analysis that was conducted through a binary logistic regression model, and variables were selected by backward selection with the elimination of the variables at *p*-value over 0.10. All *P* values lower than 0.05 were considered to be statistically significant. All analyses were performed by IBM SPSS Statistics ver. 25.0 (IBM Co. Armonk, NY, USA). This study was reviewed and approved by Seoul National University Hospital Institutional Review Board (IRB no. 1110-014-380).

## Results

### Characteristics of Desensitized Patients

A total of 1,143 procedures of desensitization of platins, taxanes, or mAbs were performed in 228 patients over 3 years; 117 patients were female (51%) and the average age was 57.5 ± 11.4 years. Frequent indications for chemotherapy were as follows: ovarian cancer (75 patients, 33%), colon cancer (28 patients, 12%), cervical cancer (16 patients, 7%), breast cancer (15 patients, 6%), and gastric cancer (14 patients, 6%). The characteristics of patients according to major drug types are described in Table 1. All initial HSRs were immediate reactions. The severity of initial HSRs was mild, moderate and severe in 11, 57, and 32% of cases, respectively. Among mAbs, rituximab accounted for 37 of 51 patients (73%) in 159 of 263 desensitization procedures (61%).

**Table 1:** Characteristics of 228 patients included in the study.

**Drug type**	**Platins (123)**	**Taxanes (54)**	**mAbs (51)**
Desensitized drug	Oxaliplatin (57), Carboplatin (49), Cisplatin (17)	Paclitaxel (42), Docetaxel (12)	Rituximab (37), Cetuximab (5), Obinutuzumab (3), Daratumumab (2), Brentuximab, Infliximab, Pembrolizumab, Pertuzumab (1)
Female (%)	94 (76.4)	54 (100)	29 (56.9)
Age (year)	58.6 ± 11.1	52.2 ± 9.9	60.6 ± 12.1
Indication (N)	Ovarian cancer (38), colon cancer (17), gastric cancer (14), cervical cancer (13), pancreatic cancer (10), colon cancer, rectal cancer (7), peritoneal Cancer (5), ovarian cancer (3), endometrial cancer (2), ampullary cancer, common bile duct cancer, diffuse large B-cell lymphoma, IPMN associated cancer, gallbladder cancer, klatskin tumor, vaginal cancer (1)	Ovarian cancer (34), breast cancer (14), cervical cancer (2), colon cancer, endometrial cancer, angiosarcoma, leiomyosarcoma (1)	Diffuse large B-cell lymphoma (10), follicular lymphoma (6), chronic lymphocytic leukemia, non-Hodgkin lymphoma (4), colon cancer (3), multiple myeloma, Waldenstrom's macroglobulinemia (2), breast cancer, cervical cancer, rectal cancer, acute lymphoblastic leukemia, burkitt lymphoma, CNS lymphoma, Crohn's disease, HCV-liver cirrhosis, Hodgkin lymphoma, gastric MALToma, idiopathic pulmonary fibrosis, lymphoblastic lymphoma, marginal zone B-cell lymphoma, mediastinal large B cell lymphoma, myasthenia gravis, polymyositis, prolymphocytic leukemia, rheumatoid arthritis, skin cancer (1)
Initial HSR cycle no., median (IQR)	9 (5–13)	1 (1–2)	1 (1–3)

*mAbs, monoclonal antibodies; HSR, hypersensitivity reaction; IQR, interquartile range*.

Among the 228 patients, 24 patients (11%) underwent skin tests before desensitization and 42% (10 out of 24 patients) had positive results. Skin test results were positive in 58.8% (10/17) of platin-reactive patients, 33.3% (1/3) of taxane-reactive patients, and 75% (3/4) of mAb-reactive patients (Supplementary Table 2).

### Outcomes of Desensitization

Out of the 1,143 procedures of desensitization, desensitization completion rate was 99% in total. BTRs occurred in 292 out of 1,143 procedures (26%). The BTR occurrence rates differed among major drug types, being 34%, 12%, and 18% for platins, taxanes, and mAbs, respectively (Table 2).

**Table 2:** Outcomes of 1,143 cases of desensitization.

**Drug type**	**Platins**	**Taxanes**	**mAbs**
No. of desensitized patients	123	54	51
No. of desensitization procedures	651	229	263
BTR occurrence rate (%)^*^	34	12	18
Completion rate (%)	99	99	97

*mAbs, monoclonal antibodies; BTR, breakthrough reaction*.

* *p < 0.001*.

**Figure 1:**
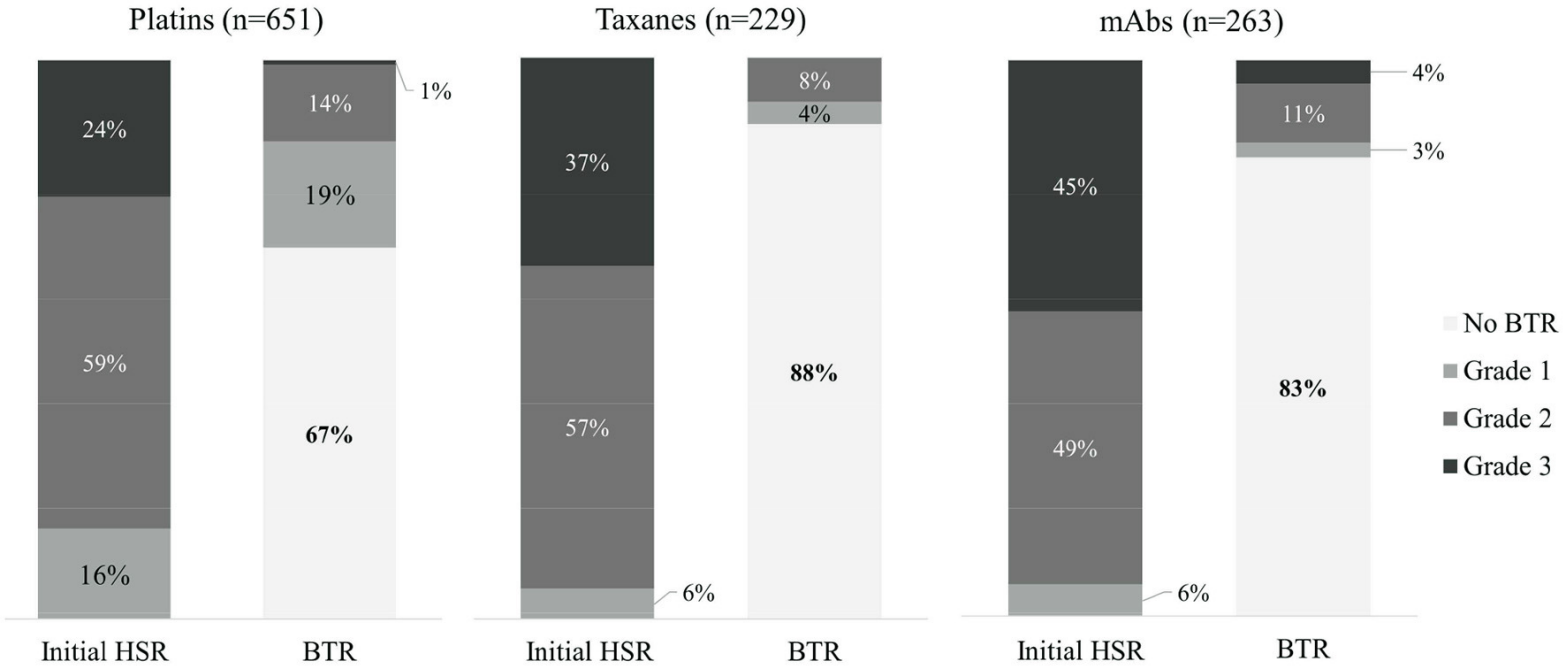
The comparison of severity of initial HSRs and BTRs in each drug type. mAbs, monoclonal antibodies; HSR, hypersensitivity reaction; BTR, breakthrough reaction.

**Figure 2:**
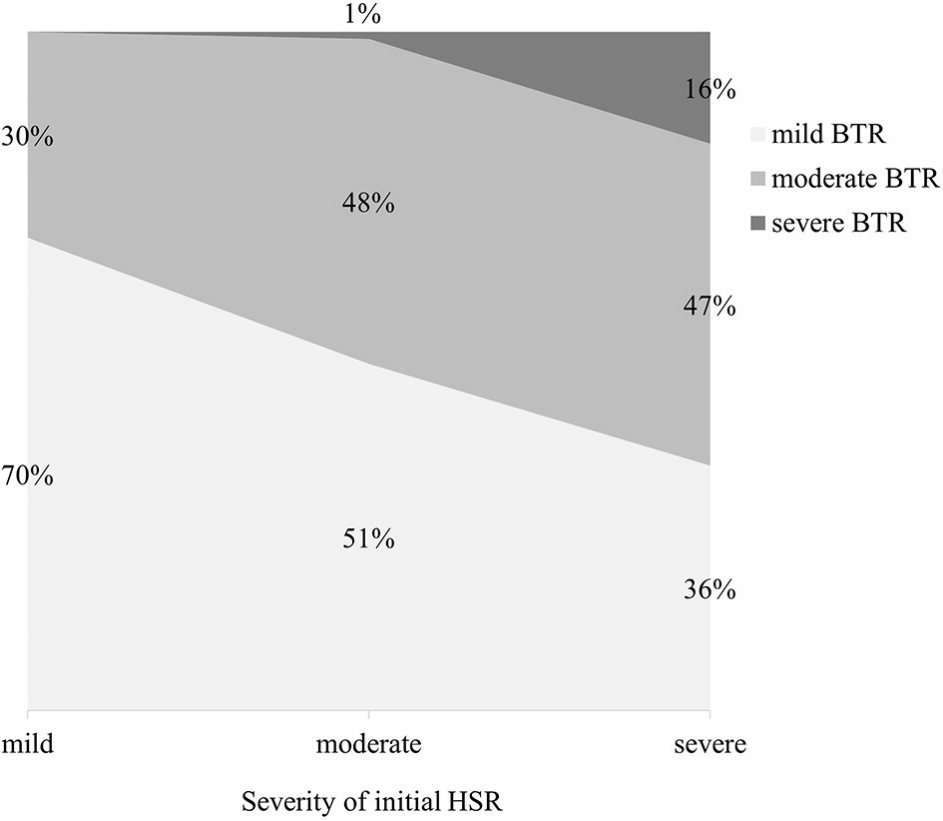
The severity of BTRs reactions according to that of initial HSRs. HSR, hypersensitivity reaction; BTR, breakthrough reaction.

Out of the 1,143 desensitization procedures, 89% of patients either had mild or no BTRs. Among the 292 desensitization procedures with BTRs, 57% presented BTRs of milder severity than the initial HSR. The severity of initial HSRs and BTRs showed differences among major drug types (Figure 1). Severe HSR was more common in mAbs. BTRs of any grade were more common in platins, but severe BTR was greatest in mAbs. In addition, while 16% of patients with severe initial HSRs also had severe BTRs, only 1% of patients with moderate initial HSRs experienced severe BTRs and no patient with mild initial HSR had severe BTR (Figure 2).

### Clinical Manifestation of Initial HSRs and BTRs

**Figure 3:**
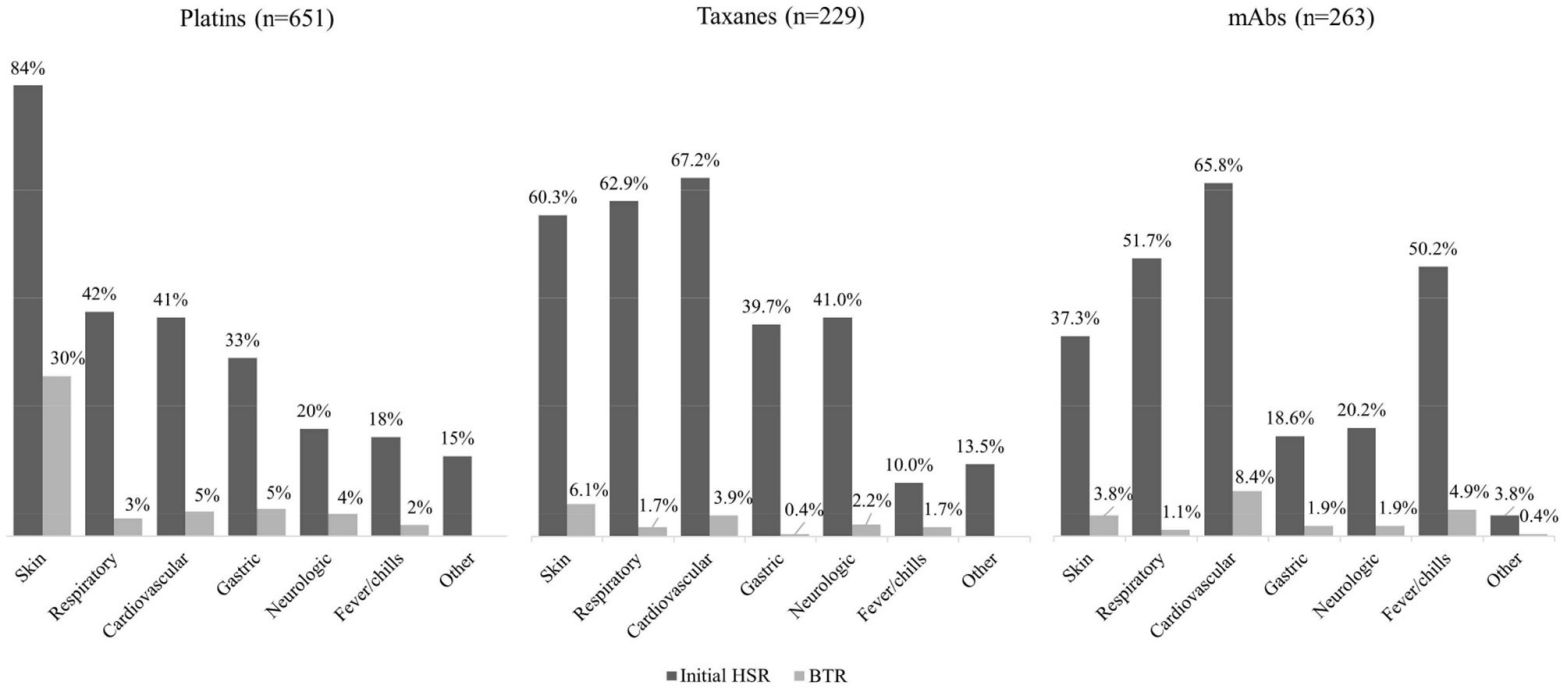
Clinical symptoms of initial HSRs and BTRs in 1,143 procedures of desensitization. mAbs, monoclonal antibodies; HSR, hypersensitivity reaction; BTR, breakthrough reaction.

The symptoms of initial HSRs and BTRs were classified according to involved organ systems as follows: skin, respiratory, cardiovascular, gastric, neurologic, fever/chills and other (anaphylaxis, whole body ache, blurry eyes, congested sensation of nose and ears, and not otherwise specified). The percentages of each classified symptoms are shown in Figure 3. For platins, symptoms involving the skin were the most frequent in both initial HSRs and BTRs. For taxanes, cardiovascular and respiratory symptoms occurred most frequently. For mAbs, cardiovascular, respiratory symptoms, and fever/chills were the most common presentations of the initial HSRs; 8% of them (11% for rituximab and 5% for the other mAbs combined) occurred solely as fever/chills without other symptoms.

**Figure 4:**
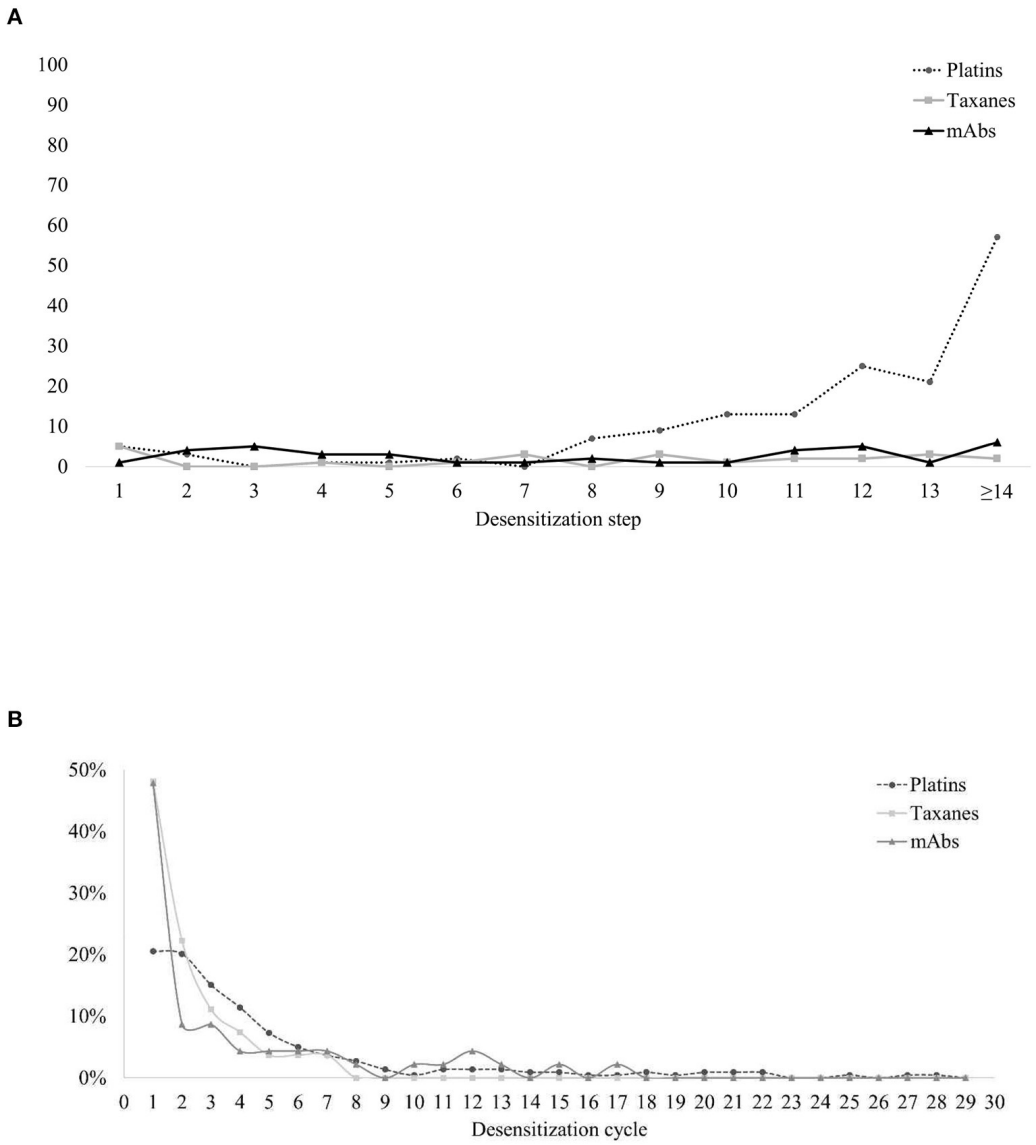
Characteristics of the occurrence of BTRs. **(A)** BTRs during desensitization: the steps in which BTRs occurred. **(B)** BTRs along the clinical course: the cycles of desensitization in which BTRs occurred. BTR, breakthrough reactions; mAbs, monoclonal antibodies.

In total, 95% of the BTRs occurred during the desensitization process, and the rest of the BTRs occurred within 1 h after the procedure. The onset of BTRs differed among the types of target drug. In taxanes and mAbs, BTRs occurred similarly throughout the steps of desensitization protocol, whereas in platins BTRs mostly appeared in the later steps (Figure 4A). The BTR occurrence rate decreased as patients went through several desensitization cycles, and 80% of the BTRs occurred within the 6th desensitization cycle (Figure 4B). For taxanes and mAbs, 48% of the BTRs occurred during the first desensitization and the occurrence rate decreased during subsequent cycles. For platins, BTR occurrence rate during the first desensitization cycle was only 21%.

### Comparison of Characteristics Between Procedures With No or Mild BTRs and Those With Moderate to Severe BTRs

The clinical characteristics were compared between desensitization procedures with no or mild BTRs and those with moderate to severe BTRs (Table 3; Supplementary Table 3). The severity of initial HSRs and the number of previous desensitizations did not differ between the two groups.

**Table 3:** Comparison of characteristics between the procedures with no or mild BTRs and those with moderate to severe BTRs.

	**No BTR, Gr 1** **(*n* = 992)**	**BTR ≥Gr 2** **(*n* = 151)**	***p* value**
Severity of initial HSR			
Grade 1	107 (11%)	7 (5%)	0.121
Grade 2	546 (55%)	89 (59%)	
Grade 3	339 (34%)	55 (36%)	
Steroid premedication	771 (78%)	96 (64%)	<0.001
Desensitization cycle	4.5 ± 5.0^*^	4.4 ± 4.8	0.893

*BTR, breakthrough reaction*.

* *Cycles in which grade 1 BTR occurred (n = 140)*.

As desensitization cycles progressed, the occurrence rate of moderate to severe BTRs was significantly reduced, and this effect was most pronounced for taxanes, although it showed statistically marginal significance (Table 4). The severity of the initial HSRs showed a significant relationship with moderate to severe BTRs for platins, whereas this association was not observed for taxanes and mAbs.

**Table 4:** Risk factors of moderate to severe BTR by logistic regression analysis.

	**Odds ratio**	**95% CI**	***p* value**
Steroid premedication			
Platins	0.504	0.311–0.818	0.006
Taxanes	-	-	-
mAbs	0.542	0.27–1.087	0.084
Desensitization cycle			
Platins	0.944	0.899–0.992	0.022
Taxanes	0.778	0.602–1.005	0.055
mAbs	0.940	0.864–1.022	0.149
Severity of initial HSR			
Platins	1.556	1.058–2.288	0.025
Taxanes	0.765	0.317–1.843	0.550
mAbs	1.124	0.606–2.086	0.710

*CI, confidence interval; mAbs, monoclonal antibodies; HSR, hypersensitivity reaction (Analyzed with the number of procedures with moderate to severe BTRs)*.

### BTR Occurrence Rate Depending on Steroid Premedication

Steroid was administered in 867 out of 1,143 (76%) desensitization procedures, mostly for antiemetic or antineoplastic purposes, not as premedication for desensitization. All desensitization for taxanes involved dexamethasone for antiemetic purposes (22). For mAbs, 51% of procedures involved steroid as a part of the chemotherapy regimen (e.g., R-CHOP). For platins, premedication with hydrocortisone was sometimes administered at the discretion of the attending physician after multiple cycles of chemotherapy, since repetitive exposure to platins has been reported to be the major risk factor for HSRs (32). In total, 95% of the patients maintained the same premedication protocol during all desensitization procedures.

The use of steroid was significantly lower in the patients with moderate to severe BTRs compared to those with no or mild BTRs (Table 3). In addition, logistic regression analysis revealed that the use of steroid was statistically significant in reducing the occurrence rate of moderate to severe BTRs, and this preventative effect was more pronounced for platins (Table 4). The odds ratio for taxanes could not be calculated as all patients received steroid for antiemetic purposes. When subdivided by the severity of initial HSRs, the association of moderate to severe BTR occurrence and steroid was statistically significant in patients whose initial HSRs were moderate to severe (Table 5).

**Table 5:** The association of moderate to severe BTR occurrence and steroid administration according to the severity of initial HSR.

	**No steroid**	**Steroid**	**OR**	**95% CI**	***p* value**
Total					
Moderate to severe BTR (%)	55/276 (20%)	96/867 (11%)	0.500	0.348–0.720	<0.001
Grade 1 initial HSR	0/13 (0%)	7/101 (7%)	-	-	1.000
Grade 2/3 initial HSR	55/263 (21%)	89/766 (12%)	0.497	0.343–0.720	<0.001
Platins					
Moderate to severe BTR (%)	31/148 (21%)	63/503 (13%)	0.540	0.336–0.870	0.010
Grade 1 initial HSR	0/8 (0%)	4/87 (5%)	-	-	1.000
Grade 2/3 initial HSR	31/140 (22%)	59/416 (14%)	0.581	0.358–0.944	0.027
mAbs					
Moderate to severe BTR (%)	24/128 (19%)	15/135 (11%)	0.542	0.270–1.087	0.081
Grade 1 initial HSR	0/5 (0%)	2/6 (33%)	-	-	0.455
Grade 2/3 initial HSR	24/123 (20%)	13/129 (10%)	0.462	0.224–0.956	0.034

*OR, odds ratio; CI, confidence interval; BTR, breakthrough reaction; HSR, hypersensitivity reaction (Analyzed with the number of procedures with moderate to severe BTRs)*.

## Discussion

This study showed the outcomes of 1,143 desensitization procedures performed to 228 patients with one-bag protocol. We report a completion rate of 99% which is comparable to previous studies.

In this study, BTR occurrence rate was 26% in total −34% in platins, 12% in taxanes, and 18% in mAbs. The incidence of BTR varies among literature as patient selection criteria and protocols are different, but the results are in line with our study. Regarding platins and taxanes, BTR occurrence rate is reported to be 35–59% and 21–30% respectively in multi-bag protocols. In one-bag protocols, it ranged between 27–61% for platins with little evidence of significant differences compared to multi-bag protocols (33). Moreover, BTR occurrence rate may be influenced by the proportion of severe HSRs included in the analysis. A previous study of 490 one-bag desensitization procedures reported a noticeably low BTR occurrence rate of 5% (12). While our study included 32% of severe initial HSRs, this study included only 11.8%.

Regarding the association between the severity of initial HSRs and that of BTRs in desensitization, our study showed that severe BTR occurred more frequently with severe initial HSRs: only 1% of patients with mild to moderate initial HSRs, but 16% for those with severe initial HSRs. Sloane et al. published a large data with 2,177 cases of multi-bag desensitization with similar results: the incidence of severe BTR was 2–3% in patients with mild to moderate initial HSRs, but 9% in those with severe initial HSRs (7). Our results suggest that if the initial HSR is mild, the chances of a future moderate to severe HSR during desensitization seem to be lower. Previous studies seem to conclude that the severity of the initial reaction is not linked necessarily to a diagnosis of allergy; however, in line with our findings, most authors seem to agree that patients with more severe initial HSRs seem to experience more severe reactions during desensitization or challenge (34). This could have implications in the risk assessment and the management pathway personalization for these patients, and further studies on different populations would be helpful.

The occurrence rate for severe initial HSRs was relatively higher in our study, but it cannot be readily interpreted as the difference between one-bag and multi-bag protocols. In addition to varying drugs and premedication between the two studies, there are ample reports that one-bag protocol is not inferior to multi-bag. Sala-Cunill et al. showed equivalent outcomes of efficacy (99% of success rate), tolerability, and safety in one-bag desensitization protocol from a 5-year experience (8). We also have previously reported that the efficacy and safety of one-bag desensitization protocol is not inferior to multi-bag protocol in taxane desensitization (14–16).

The incidence of BTRs differed according to the type of drugs. However, we cannot directly compare the BTR rates since the proportion of steroid premedication differed profoundly among the drugs. For example, taxanes presented with low risk of BTRs, but steroid was administered in all procedures, which might have underestimated the incidence of BTRs.

The onset of the initial HSR differed among major drug types: the ninth cycle was the median for platins and the first cycle for taxanes and mAbs. These results are in accordance with previous studies which explain the mechanisms of HSR upon different types of drugs. In case of platins, HSRs are mostly acquired through immunological sensitization, thus it is known that the incidence of HSRs increases with multiple exposures (33). The majority of HSRs to taxanes are thought to be determined by the direct activation of complement system by the drugs, which explains that most HSRs occur with the first or second doses (4).

The symptoms of initial HSRs showed different patterns among major drug types. For platins, skin symptoms were the most common; for taxanes, skin and cardiovascular symptoms were most observed; for mAbs, cardiovascular symptoms and fever/chills occurred most frequently. These findings are consistent with existing literature (35). The different mechanisms behind HSRs, such as IgE-mediated reactions and cytokine release, are thought to be responsible for the differences in clinical presentation (35). However, regarding mAbs, rituximab accounted for 60.5% of mAb desensitization procedures in this study. This might have skewed the composition of symptoms in the initial HSRs and BTRs of mAbs, as rituximab is known to cause fever/chills frequently (36). In our study, fever and chill as initial HSR developed in 59.1% of rituximab and 36.5% of the other mAbs combined.

Studies on the risk factors for hypersensitivity are largely divided into studies that analyze risk factors for the occurrence of HSRs in patients treated with chemotherapy and studies that analyze risk factors for the occurrence of BTRs in patients who undergo desensitization. As for initial HSRs, a study that included 162 patients who received oxaliplatin desensitization in Japan performed logistic regression analysis which showed that sex and blood eosinophil count are related with oxaliplatin HSRs (17). A positive skin test and a history of drug allergy are also reported to be useful in the risk stratification process for drug hypersensitivity (5, 19, 21, 23). As for BTRs, atopy and positive skin prick test are reported to be the risk factors (23, 37). A Korean study with 234 procedures of desensitization in 58 patients reported the previous exposure to high doses of culprit antineoplastic agent as a risk factor of BTR occurrence (22). Moreover, in a study with 1,471 desensitizations to 272 patients, a total IgE > 100 kU/L and over 10 previous administrations of platins were also reported as risk factors for BTR during platin desensitization (1). On this background, our study suggests an additional risk factor for moderate to severe BTR: the severity of initial HSR, especially in platins. Based on this result, it can be considered to incorporate the severity of initial HSR in deciding the initiation of platin desensitization. However, more prospective studies with larger sample sizes are needed to evaluate whether this association can be replicated in desensitization with other drugs.

The trends of BTRs along the desensitization process have been well-studied. In a study that included 189 cases of desensitization in 23 patients, 47.8% of the patients experienced BTRs on their first desensitization trial (38). A study with 413 desensitization cases also reported that 61% of the BTRs occurred during either the first or second desensitization cycles, and the frequency and severity of BTRs decreased over the course of subsequent desensitization process (6). In addition, there has been an observation that BTRs to taxanes decline with subsequent exposures (37). Such results are in accordance with the results of this study which also showed that most BTRs occurred during the first desensitization cycle. Desensitization is thought to allow for the temporary tolerance to the drug (5, 7) but recent studies revealed the possibility of attaining long-term tolerance or immunological anergy after repeated desensitization by the induction of IL-35 or regulatory T cells (39, 40). For HSRs to mAbs, cytokine-release related reactions are frequent, and the symptoms are known to be more severe with higher tumor burdens (35). If patients can continue the first-line treatment with the help of desensitization, the response to treatment and overall decrease in tumor burden may lead to a lower rate of BTR occurrence. Thus, it may be plausible to continue the attempts for desensitization even if BTRs occur, as we can expect these symptoms to alleviate as the cycle proceeds.

Studies on the effect of premedication in desensitization are scarce. Routine premedication with antihistamines and steroids is often used to prevent BTRs (29). Findings of a previous study suggest that steroid premedication can be considered if the BTR symptom resembles a reaction caused by cytokine release (41). A Japanese study using a 3-bag protocol for oxaliplatin proposed an additional administration of premedication before the third bag based on the possibility of a BTR occurring during the last phase of the protocol (42). A study involving 155 paclitaxel desensitization cases analyzed the effects of antihistamine and steroid premedication, and the results showed no differences in BTR occurrence between the groups who did and did not receive premedication (43). These results could not be directly compared to our study since patients with taxane desensitization in our study all received steroids as part of their chemotherapy regimen. Thus, additional studies are required with comparable study subjects.

The results of this study showed that, in this population, there seem to be statistically significant differences in the occurrence of moderate to severe BTRs depending on the absence or presence of steroid administration. For mAbs, there was a marginal statistical significance for this same effect. This statistical insignificance of mAbs might be explained by the small number of procedures or the heterogeneity of the drugs. Even if association does not imply causation, we wonder if this could potentially mean that corticosteroid premedication might be effective to reduce the occurrence rate of BTRs during desensitization in patients with moderate to severe initial HSRs. However, previous articles showed data going against this argument (26), so further specifically-designed prospective studies will be needed to answer this question.

This study has some limitations. Firstly, there is insufficient allergy workup as per guidelines (skin testing, *in vitro* biomarkers, and drug challenge), which could incur selection bias. Namely, the lack of confirmatory tests might involve that a percentage of patients might not be hypersensitive, and the lack of patient endophenotyping (e.g., into type I hypersensitivity, cytokine release syndrome, or mixed reactions) might further confuse the results, as the response to desensitization and to premedication differs among endophenotypes. Secondly, given the study design and local guidelines for the administration of antineoplastics, premedication with corticosteroids was heterogeneous and not randomized, case-control groups were not matched, and there was no sample size or power calculation.

In conclusion, most patients with HSRs to antineoplastic agents and biologics may safely receive chemotherapy through a one-bag desensitization protocol. Further studies on each drug with larger sample size can help verify the risk factors of BTRs and evaluate the efficacy of steroid premedication in improving the safety of desensitization in high-risk patients.

## Data Availability Statement

The original contributions presented in the study are included in the article/Supplementary Material. Further inquiries can be directed to the corresponding author.

## Ethics Statement

The studies involving human participants were reviewed and approved by Seoul National University Hospital Institutional Review Board (IRB no. 1110-014-380). Written informed consent for participation was not required for this study in accordance with the national legislation and the institutional requirements.

## Author Contributions

HHK: conceptualization, data curation, and writing – original draft. JS and YHA: writing – original draft and editing. HK: data curation, writing – review and editing. J-EY, JHS, and SYL: conceptualization and data curation. DYK: data curation and statistical work. H-RK: conceptualization, writing – original draft and editing. All authors contributed to the article and approved the submitted version.

## Funding

This research was supported by a grant of the Korea Health Technology R&D Project through the Korea Health Industry Development Institute (KHIDI), funded by the Ministry of Health and Welfare, Republic of Korea (grant number: HI21C1372).

## Conflict of Interest

The authors declare that the research was conducted in the absence of any commercial or financial relationships that could be construed as a potential conflict of interest.

## Publisher's Note

All claims expressed in this article are solely those of the authors and do not necessarily represent those of their affiliated organizations, or those of the publisher, the editors and the reviewers. Any product that may be evaluated in this article, or claim that may be made by its manufacturer, is not guaranteed or endorsed by the publisher.
