# Supplementary material for: Analysis of Breakthrough Reactions in 1,143 Desensitization Procedures in a Single Tertiary Hospital Using a One-Bag Desensitization Protocol
Source: Front Allergy. 2022 Feb 11;3:786822. doi: 10.3389/falgy.2022.786822 (PMC8974795; doi:10.3389/falgy.2022.786822)
Supplement: Supplementary file 1 [file Table_1.docx]

**Supplement material 1** An example of a 1-bag 12-step desensitization protocol for oxaliplatin 100 mg

| Step | Rate (mL/hr) | Time (min) | Dose (mg) | Volume (mL) |
| --- | --- | --- | --- | --- |
| 1 | 0.1 | 15 | 0.0114 | 0.025 |
| 2 | 0.2 | 15 | 0.0227 | 0.05 |
| 3 | 0.5 | 15 | 0.0568 | 0.1 |
| 4 | 1.2 | 15 | 0.1364 | 0.2 |
| 5 | 2.5 | 15 | 0.2841 | 0.4 |
| 6 | 5 | 15 | 0.5682 | 0.75 |
| 7 | 12.5 | 15 | 1.4205 | 1.5 |
| 8 | 25 | 15 | 2.8409 | 3.125 |
| 9 | 50 | 15 | 5.6818 | 6.25 |
| 10 | 90 | 15 | 10.2273 | 12.5 |
| 11 | 150 | 15 | 17.0455 | 25 |
| 12 | 250 | 32.6 | 61.7045 | 160.1 |
| Oxaliplatin 100 mg/20 mL was reconstituted with 200 mL of 5% dextrose water. The concentration of the solution was 0.45455 mg/mL.  Dose (mg) = Rate (mL/hr) × time/60 (hr) × concentration (mg/mL) | | | | |
