# Supplementary material for: Analysis of Breakthrough Reactions in 1,143 Desensitization Procedures in a Single Tertiary Hospital Using a One-Bag Desensitization Protocol
Source: Front Allergy. 2022 Feb 11;3:786822. doi: 10.3389/falgy.2022.786822 (PMC8974795; doi:10.3389/falgy.2022.786822)
Supplement: Supplementary file 2 [file Table_2.docx]

**Supplement material 2** Skin testing for each comparison of characteristics between patients with no or mild BTRs and moderate to severe BTRs in different drug types

| **Drug type** | **Platins (123)** | **Taxanes (54)** | **mAbs (51)** |
| --- | --- | --- | --- |
| Concentration for skin prick test | Oxaliplatin: 5 mg/mL  Carboplatin 10 mg/mL  Cisplatin 0.5 mg/mL | Paclitaxel 6 mg/mL  Docetaxel 2 mg/mL | Rituximab 10 mg/mL  Cetuximab 5 mg/mL  Obitunuzumab 25 mg/mL |
| Skin test positivity (numbers among patients tested) | 10 / 17 | 1 / 3 | 3 / 4 |
