# Supplementary material for: Analysis of Breakthrough Reactions in 1,143 Desensitization Procedures in a Single Tertiary Hospital Using a One-Bag Desensitization Protocol
Source: Front Allergy. 2022 Feb 11;3:786822. doi: 10.3389/falgy.2022.786822 (PMC8974795; doi:10.3389/falgy.2022.786822)
Supplement: Supplementary file 3 [file Table_3.docx]

**Supplement material 3** Comparison of characteristics between patients with no or mild BTRs and moderate to severe BTRs in different drug types

|  | Platins | | | Taxanes | | | mAbs | | |
| --- | --- | --- | --- | --- | --- | --- | --- | --- | --- |
|  | No BTR, Gr 1  (n=557) | BTR ≥ Gr 2  (n=94) | *p* value | No BTR, Gr 1  (n=211) | BTR ≥ Gr 2  (n=18) | *p* value | No BTR, Gr 1  (n=224) | BTR ≥ Gr 2  (n=39) | *p* value |
| Severity of initial HSR |  |  |  |  |  |  |  |  |  |
| Grade 1 | 76 (18%) | 4 (4%) | 0.008 | 6 (3%) | 1 (6%) | 0.443 | 9 (4%) | 2 (5%) | 0.922 |
| Grade 2 | 260 (60%) | 64 (68%) |  | 113 (56%) | 11 (61%) |  | 80 (37%) | 14 (36%) |  |
| Grade 3 | 97 (22%) | 26 (28%) |  | 83 (41%) | 6 (33%) |  | 128 (59%) | 23 (59%) |  |
| Steroid premedication | 332 (77%) | 63 (67%) | 0.050 | 211 (100%) | 18 (100%) | - | 114 (53%) | 15 (39%) | 0.074 |
| *BTR; breakthrough reaction, mAbs; monoclonal antibodies* | | | | | | | | | |
